# Comparative profiling of T cell and macrophage subsets in cutaneous squamous cell carcinoma and basal cell carcinoma

**DOI:** 10.1038/s41598-025-22486-1

**Published:** 2025-10-09

**Authors:** Linus Winter, Jutta Ries, Christoph Vogl, Leah Trumet, Carol Immanuel Geppert, Carina Scholtysek, Manuel Olmos, Marco Kesting, Manuel Weber

**Affiliations:** 1 Department of Oral and Cranio-Maxillofacial Surgery, Uniklinikum Erlangen Friedrich-Alexander University, Erlangen-Nürnberg, FAU Glückstraße 11, Erlangen, Germany; 2 Deutsches Zentrum Immuntherapie (DZI), University of Erlangen-Nuremberg and Universitätsklinikum Erlangen, Erlangen, Germany; 3 Comprehensive Cancer Center Erlangen-EMN (CCC ER-EMN), Erlangen, Germany; 4 Comprehensive Cancer Center Alliance WERA (CCC WERA), Erlangen, Germany; 5 Bavarian Cancer Research Center (BZKF), Erlangen, Germany; 6 Department of Operative Dentistry and Periodontology, Friedrich-Alexander Universität Erlangen-Nürnberg (FAU), Erlangen, Germany; 7 Institute of Pathology, University Hospital Erlangen, Friedrich-Alexander University, Erlangen-Nürnberg, Erlangen, Germany; 8 Department of Internal Medicine 3, University of Erlangen-Nuremberg and Universitätsklinikum Erlangen, Erlangen, Germany

**Keywords:** NMSC, cSCC, BCC, Macrophages, T-cells, Cancer, Skin cancer, Tumour immunology

## Abstract

The yearly incidence of basal cell carcinoma (BCC) and cutaneous squamous cell carcinoma (cSCC) continues to rise in the ongoing skin cancer epidemic. Mousa and colleagues have previously highlighted the crucial role of the immune system in the development and progression of both tumor types. Therefore, immune cells in close proximity to, or even infiltrating, the tumor should be of particular interest. Previously, we demonstrated differential expression of immune checkpoint markers, including both activating and inhibitory markers, between BCC and cSCC. The present study aims to further investigate the tumor microenvironment, with a focus on T cells, macrophages, and dendritic cells, among others. Furthermore, we analyzed possible associations between cSCC marker expression and clinicopathological data. In order to evaluate the expression levels and topographic distribution profiles of CD4, CD8, Foxp3, CD68, CD163, and CD11c, tissue microarrays of the invasive front as well as the tumor core of BCC and cSCC samples were analyzed. Chromogenic immunohistochemistry was performed, and the labeling index was determined using QuPath. Stroma cell labeling indices of CD4**, CD8*, Foxp3*, CD68*, CD163**, and CD11c* were significantly higher in both the tumor core and the invasive front of cSCC samples as compared to BCC (*p < 0.001; **p < 0.050). In cSCC, the ratios of CD163/CD68 (*p* < 0.001) and CD163/CD11c (*p* = 0.001) were markedly elevated. The invasive front of both tumor entities showed higher expression levels of all immune markers compared to the tumor core, when significant levels were reached. Lastly, an association between cSCC sLI CD4 and T status (*p* = 0.011), CD11c and N status (*p* = 0.041), CD68 and infiltrations depth (*p* = 0.018), as well as CD8 and CD11c and previous tumor (*p* = 0.006; *p* = 0.029, respectively) was observed. This study provides a comprehensive analysis of the tumor microenvironment (TME) of BCC and cSCC, emphasizing the distinct expression patterns of the TME with regard to T cell and macrophage-associated markers. The cSCC TME exhibited higher expression levels of all investigated markers, predominantly at the invasive front rather than the tumor core. Further, more comprehensive analysis of these TMEs is required to understand their impact on clinicopathological data, response to immunotherapy and predictive ability.

**Supplementary Information:**

The online version contains supplementary material available at 10.1038/s41598-025-22486-1.

## Introduction

Non-melanoma skin cancer (NMSC) in combination with cutaneous melanoma has the highest number of cases of any human malignancy^1,2^. In 2017 alone, 7.7 million incident cases of NMSC were recorded worldwide, of which 5.9 million cases were due to basal cell carcinoma (BCC) and 1.8 million due to cutaneous squamous cell carcinoma (cSCC)^3^. Both BCC and cSCC show increasing incidence and mortality rates of skin cancer^4^. With the incidence of BCC steadily rising at roughly 5% annually and the cSCC incidence is soon to match^5^, the ongoing epidemic of skin cancer is far from over. Due to a population with high UV exposure, the increase in annual incidence is expected to continue until at least 2040^6^. Other risk factors besides UV exposure include fair skin type, previous BCC, and genetic predisposition among others^7,8^. Regarding the predominant site of BCC and cSCC development, both are most commonly found in the head and neck region of patients, with 73% and 64% of all BCC and cSCC, respectively, located in the face^9^.

In both entities the immune system appears to play a vital role in the development and progression of BCC and cSCC, especially, in immunocompromised and elderly individuals^8^. Hence, immune cells adjacent to, or surrounding the tumor cells should be of special interest. They represent the frontline of interaction between the host’s defense and the cancer cells. The unique composition of cells surrounding and feeding the tumor cells can be collectively described as the tumor microenvironment (TME). The TME typically consists of immune cells, stromal cells, blood vessels, and extracellular matrix, but may vary between tumor entities^10^. Immune cells within the TME can exert different properties leading to either a suppressive or supportive role in cancer growth^10^.

Our group’s previous research has already highlighted the expression patterns of activating and inhibitory checkpoints, such as PD-1/PD-L1 and CD28/CD86, in NMSC, as well as a small subset of T cell and macrophage markers^11,12^. The checkpoint markers investigated in our previous work can be expressed on the cell surface of various cell types, including T cells, macrophages, and different subsets of dendritic cells^13–16^. To provide a more comprehensive overview of the findings from these earlier studies, the present study aims to examine the specific underlying cell types that were previously investigated.

Hence, within the T cell spectrum, T cell receptor affinity can be coupled with either MHC-1 or MHC-2 affinity^17^. Cytotoxic T cells (CD8+) demonstrate affinity for MHC-1, whereas CD4 + T cells show affinity for MHC-2^17^. MHC-1-mediated antigen presentation can be performed by nucleated cells, whereas MHC-2 antigen presentation is a function reserved for antigen-presenting cells, such as macrophages^17^. CD8 + T cells have been demonstrated to play a role in the destruction of cancer cells and the prevention of angiogenesis^10^. Moreover, CD8 + T cells are regarded as the most efficacious effectors in the anticancer immune response^18^. CD4 + T cells and their subtypes on the other hand, take in various roles within the TME^10^. Dependent on their differentiation they can either facilitate CD8 + cell-mediated tumor destruction as T-helper cells, or directly suppress an antitumor response, as regulatory T cells (Tregs)^10^. Tregs can be identified by their expression of forkhead transcription factor 3 (Foxp3), which is predominantly expressed by a subset of CD4 + T cells^19^.

Regarding macrophages within the TME, two phenotypes merit particular attention. Namely the M1-like macrophages and the M2-like macrophages. M1-like macrophages take in a pro-inflammatory role and hinder tumor growth, whereas M2-like macrophages are considered anti-inflammatory and, as a consequence, facilitate tumor growth and immune cell suppression^10^. CD68 may be utilized to characterize both M1-like and M2-like macrophages^20^. CD11c, which is a transmembrane protein expressed on monocytes, granulocytes, dendritic cells, and macrophages^21^, may be used to distinguish M1-like macrophages^22^. Additionally, M2-like macrophages can be distinguished by the presence of both CD68 and CD163^20^.

The objective of this study was to gain a more profound understanding of the TME of both basal cell carcinoma (BCC) and cutaneous squamous cell carcinoma (cSCC) by comprehensively reviewing the expression of specific T cell- (CD4, CD8, Foxp3) and macrophage- (CD68, CD11c, CD163) related markers. This approach was taken with the aim of elucidating the role of these cells and markers in their respective TMEs. Moreover, the objective was to investigate potential discrepancies in the spatial organization between the invasive front and the tumor core, as distinct tumor compartments, and to contrast the respective TME of BCC and cSCC patients. To visualize the immune cells of interest, antibodies were employed in a chromogenic immunohistochemical (IHC) approach using tissue microarrays (TMAs).

## Materials and methods

### Patient collective

The study was performed in accordance with the Declaration of Helsinki and received ethical approval by the local ethics comity of the Friedrich-Alexander University Erlangen-Nuremberg (Case 54_17Bc). In total the cohort comprised of 97 BCC and 105 cSCC cases, which presented in 57 woman and 103 men, whilst several patients suffered from multiple BCC/cSCC. In Table 1. demographic and histomorphological data of the investigated NMSC cases are summarized.

**Table 1:** Description of patient collective.

		BCC	cSCC
		*n*	*n*
Total number of patients	Women	57	
	Men	103	
Total number of cases		97	105
	Women	35	36
	Men	62	69
Immunocompromised		1	10
Mean age [years]	Women	76.40 ± 13.68	82.88 ± 12.79
	Men	71.14 ± 13.70	78.30 ± 8.89
pT	T1	48	47
	T2	4	21
	T3	2	22
	T4	2	1
	TX	41	14
pN	N0	17	42
	N1	0	4
	N2	0	3
	N3	0	2
	NX	80	54
pM	M0	17	63
	M1	0	2
	MX	80	40
Grading	G1	0	19
	G2	0	44
	G3	0	37
	GX	97	5
Infiltration depth [mm]	Mean	3.76 ± 4.13	6.84 ± 6.72
	Max	17.00	30.00
	Min	0.05	1.00

Values represent the number of cases by n in basal cell carcinoma (BCC) and cutaneous squamous cell carcinoma (cSCC). pT: pathological assessment of the primary tumor size/extent; pN: pathological assessment of regional lymph node involvement; pM: pathological assessment of distant metastasis. Gx = grade cannot be assessed; G1 = well differentiated; G2 = moderately differentiated; G3 = poorly differentiated.

### Sampling of tissue

#### Tissue harvesting

This retrospective analysis encompasses patient cases treated at the Department of Oral and Cranio-Maxillofacial Surgery, University Hospital Erlangen, Germany, between 2010 and 2020. To select cases, histopathological reports from the Institute of Pathology were screened, and all samples were reviewed and selected from representative H&E stains under light microscopy (Axio Imager 2, Carl Zeiss, Germany) to ensure high-quality specimens. This process was supervised by senior pathologist from the Institute of Pathology at the University Hospital Erlangen. Thereafter, TMA construction was performed as previously described by our workgroup^11^.

#### Immunohistochemical staining

**Fig. 1:**
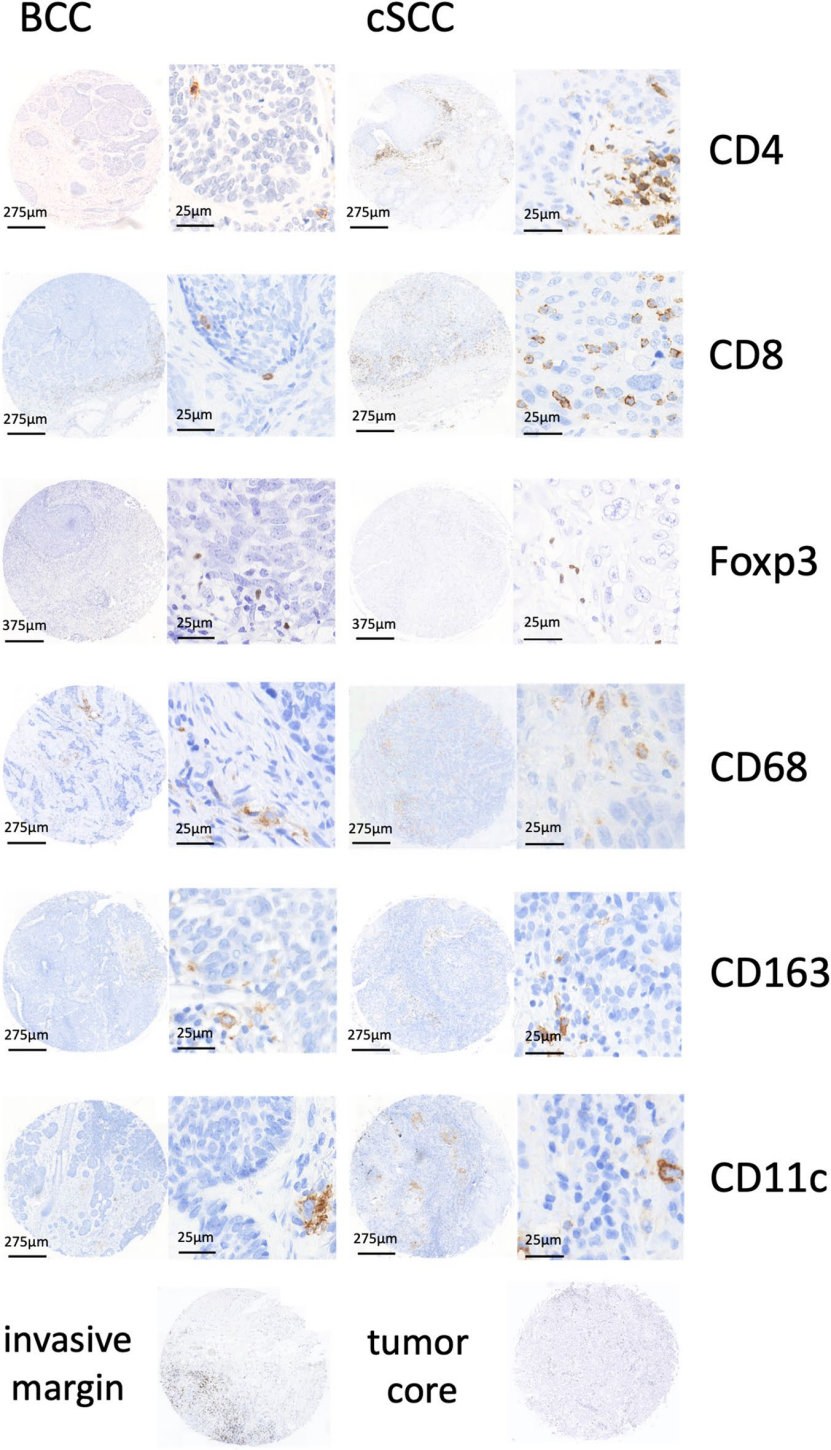
Representative TMA-cores of CD4, CD8, Foxp3, CD68, CD163, and CD11c stainings for BCC and cSCC, as well as representative invasive margin and tumor core TMA cores. Sample tissue-microarray (TMA) cores, taken as representaives, from tumor core slides of basal cell carcinoma (BCC) and cutaneous squamous cell carcinoma (cSCC) patient cases.

Slide preparation and immunohistochemical staining was performed, as previously described by our workgroup^11,23,24^. As primary antibodies, CD4 (AB133616; Abcam, Cambridge, UK; dilution 1:500), CD8 (M7130; Agilent Dako, Santa Clara, USA; dilution 1:500), Foxp3 (AB20034; Abcam, Cambridge, UK; dilution 1:750), CD68 (M0814; Agilent Dako, Santa Clara, USA; dilution 1:3000), CD163 (NCL-L-CD163; Leica Biosystems, Wetzlar, Germany; dilution 1:100), and CD11c (AB52632; Abcam, Cambridge, UK; dilution 1:750) were utilized. Further, the Histofine Simple Stain MAX PO staining kit (DAB kit, Medac, Wedel, Germany) in combination with the CD4, CD8, Foxp3, CD68, CD163, and CD11c primary antibodies were used for visualization purposes, in accordance with the instructions provided by Medac. To ensure the quality of each run, positive and negative controls were used. The positive controls were human tonsil and lymph node tissue, while the negative control was human oral mucosa. Representative tumor cores from several stains are presented in Fig. 1.

#### Digitalization and statistical analysis

**Fig. 2:**
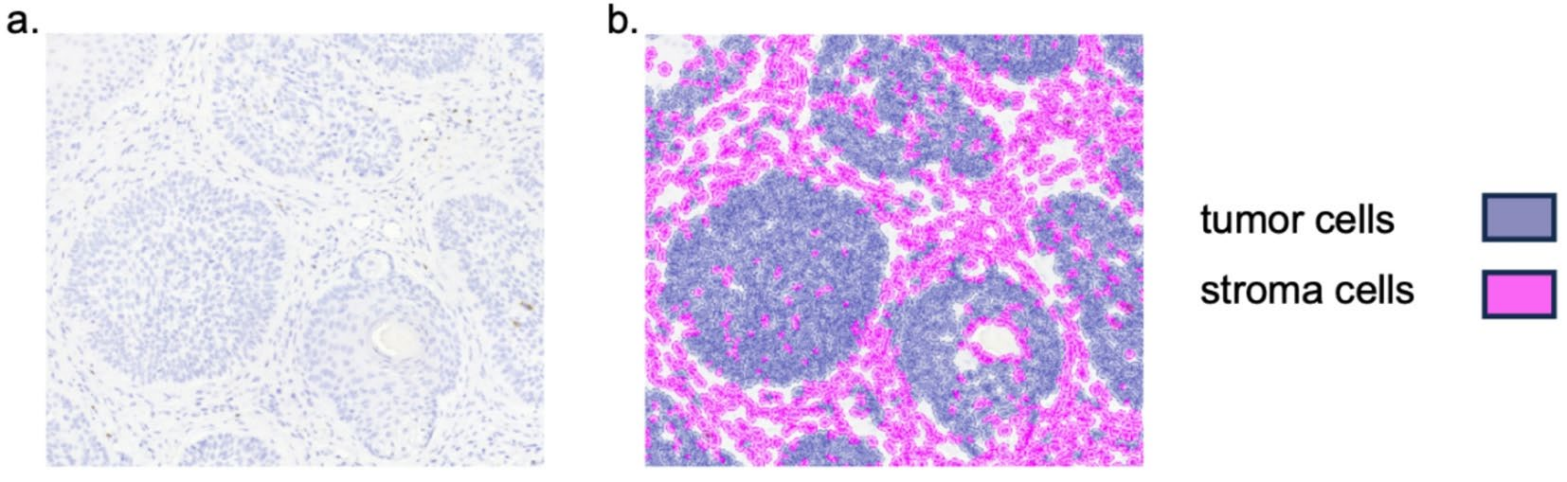
Representation of TMA core tissue segmentation. (a) Sample tissue -microarray (TMA) core of basal cell carcinoma. (b) Colored tissue segmentation overlaying the TMA core. Tissue was separated into tumor and stromal tissue as displayed.

For digitalization of IHC-stained sections all slides were scanned in collaboration with the Digital Pathology Core Unit of the Institute of Pathology at the University Hospital Erlangen (Pannoramic 250 Flash III, 3D Histech, Budapest, Hungary). As previously described by our laboratory^11^, digital quantification and analysis were performed using QuPath-0.5.1, an open-source software for bioimage analysis^25^. Figure 2 represents a sample core, after digital quantification analysis. Automated cell counting of positive and negative cells was then performed over the entire available tissue area. The data were analyzed as previously described, by relating the total number of positive cells to the number of cells detected in a given compartment as stroma or total cell labeling index. The stroma labeling index (sLI) was calculated by dividing the number of positively stained stromal cells by the total number of stromal cells in the TMA. The total cell labeling index (LI) was calculated by dividing the total number of positively stained cells by the total number of all cells in the TMA^11^. The box plot diagrams represent the median, interquartile range, minimum (min) and maximum (max). Two-sided adjusted p-values ≤ 0.05 were statistically significant; ≤ 0.001 were highly significant. Data analyses was performed using the Mann–Whitney–U-Test with IBM SPSS Statistics Version 24 (Released 2016, IBM SPSS Statistics for Windows, IBM Corp., Armonk, NY). Distribution of immune cell infiltration in different tumor compartments was compared between BCC and cSCC using LI and sLI. Ratios of marker pairings were analyzed by dividing their respective LI for BCC and cSCC. In addition, CD4, CD8, Foxp3, CD68, CD163, CD11c expressions were correlated separately for BCC and cSCC. A Spearman’s correlation test was performed. Statistical significance was reached for p-values at either the *p* ≤ 0.05 (*), or the *p* ≤ 0.01 (**) level. For Rho (ρ), either moderate positive correlation was attributed for ρ between 0.40 and 0.59, or strong positive correlation was attributed for ρ between 0.60 and 0.79^26^. The supplementary material section contains the Spearman correlation coefficient values and two-sided adjusted p-values in the Supplementary Tables 1 and 2.

## Results

### Clinicopathological results

The gender distribution was comparable between the BCC and cSCC cohorts: 35 and 36 women and 62 and 69 men. BCC and cSCC patients were similar in age. TNM staging was performed in accordance with the 2017 UICC classification^27^. Most cases were T1 carcinomas: 48 BCC (49.5%) and 47 cSCC (44.8%). No BCC showed lymph node infiltration, while 9% of cSCC did. No cases of distant metastasis were presented in the BCC cohort. However, two cases of cSCC (1.7%) developed distant metastasis. No BCC cases underwent grading. In contrast, 19 cSCC cases were graded as G1 (18.1%), 44 as G2 (41.9%), and 37 as G3 (35.2%). Further detailed information on the cohort’s demographics is provided in Table 1.

### Distribution of immune cell infiltration in different tumor compartments

#### CD4, CD8, Foxp3, CD68, CD163, CD11c expression in the invasive front and tumor core in BCC and cSCC

**Fig. 3:**
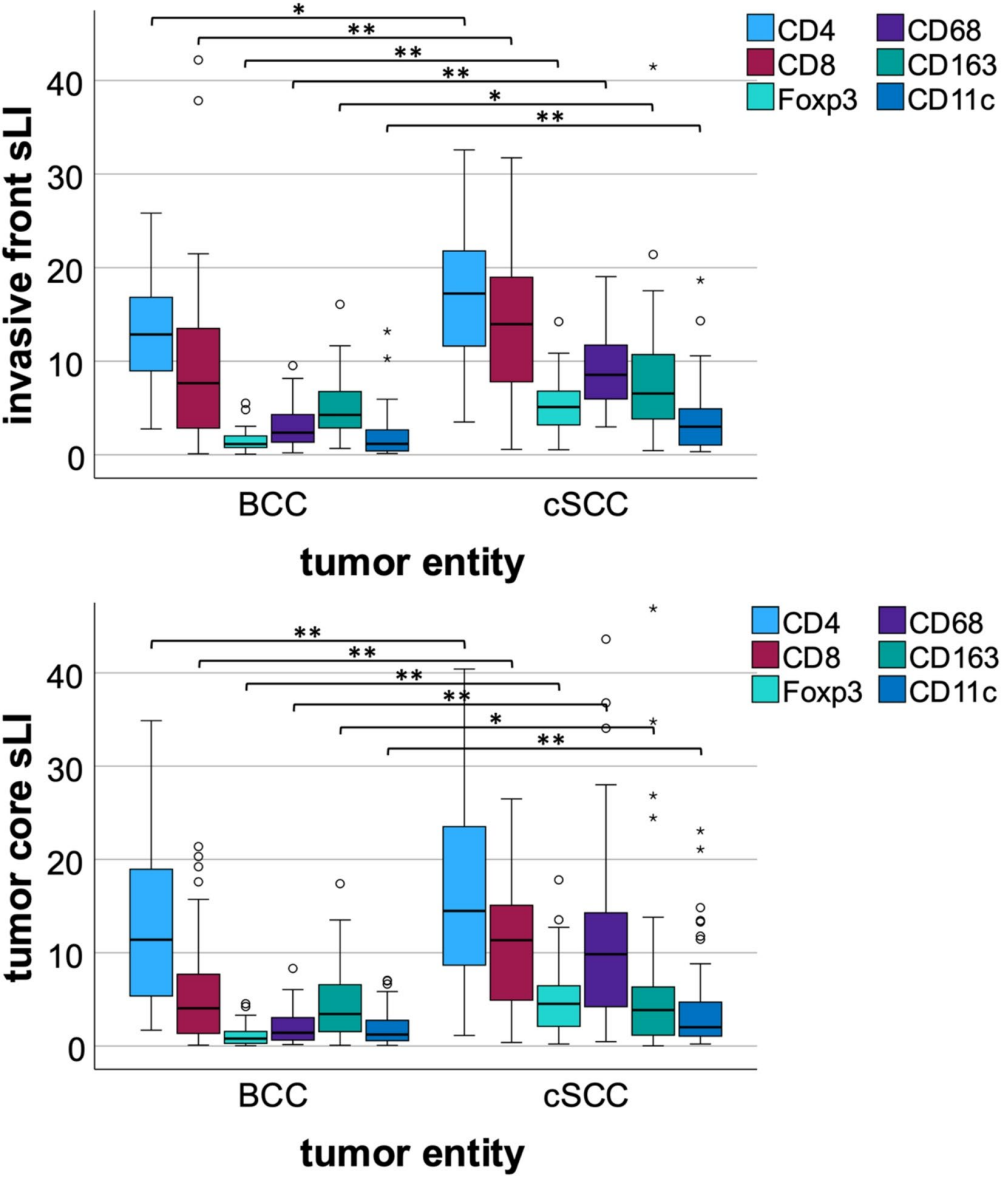
The box plots show CD4, CD8, Foxp3, CD68, CD163, CD11c expression in basal cell carcinoma (BCC) and cutaneous squamous cell carcinoma (cSCC). Boxplots represent CD4, CD8, Foxp3, CD68, CD163, CD11c expression in the invasive front and the tumor core. The tumor core is comprised of mostly epithelial tissue with some stromal tissue, whereas the invasive front represents the transition zone from tumor to stroma tissue. Stroma labeling indices (sLI) contain cell counts of stromal cells within the invasive front or the tumor core. Indices are expressed as a percentage. * = *p* < 0.050; ** = *p* ≤ 0.001. P-values were generated using the Mann-Whitney U test.

CD4 LI, and sLI demonstrated significantly elevated expression in all compartments of cSCC, as compared to BCC (p < 0.05; Table 2; Figure 3. The differences in CD8 expression between cSCC, and BCC were highly significant for the invasive front sLI, tumor core sLI, and tumor core LI (p < 0.001; Table 2; Fig. 3), as well as significant for the invasive front LI (p = 0.008; Table 2). As observed for the CD68 LI and sLI, Foxp3 also demonstrated a statistically significant increase in expression levels across both the tumor core and the invasive front of cSCC when compared to BCC LI and sLI (p < 0.001; Table 2; Fig. 3). CD163 values were found to be significantly elevated in cSCC for the invasive front LI/sLI, and tumor core sLI (p < 0.05; Table 2; Fig. 3). CD11c LI, and sLI showed significantly higher values in the invasive front of cSCC (p < 0.001, p = 0.002, respectively; Table 2; Fig. 3), as well as the tumor core (p < 0.001; Table 2; Fig. 3) favoring cSCC instead of BCC.

**Table 2:** Comparison of CD4, CD8, Foxp3, CD68, CD163, and CD11c mean, SD and p-values between BCC and cSCC in different tumor compartments by labeling indices.

		Invasive front stroma labeling index		Invasive front total cell labeling index		Tumor core stroma labeling index		Tumor core total cell labeling index	
Marker		BCC	Cscc	BCC	Cscc	BCC	Cscc	BCC	Cscc
*CD4*	n	79	51	79	51	80	87	80	87
	Mean	15.15	18.01	10.28	13.60	12.77	17.99	7.87	11.53
	SD ±	8.6	7.60	7.57	7.14	9.65	10.90	7.47	9.47
	p-value	*p* = 0.017		*p* = 0.004		*p* = 0.001		*p* = 0.003	
*CD8*	n	70	49	70	49	73	83	73	83
	Mean	9.17	14.06	6.80	9.01	5.82	11.27	3.19	6.36
	SD ±	9.70	8.23	8.22	6.72	6.25	9.13	3.94	7.11
	p-value	*p* < 0.001		*p* = 0.008		*p* < 0.001		*p* < 0.001	
*Foxp3*	n	68	48	68	48	60	91	60	91
	Mean	1.48	5.29	0.88	2.54	1.14	5.18	0.68	2.41
	SD ±	1.31	3.29	0.78	1.69	1.17	4.09	0.82	2.11
	p-value	*p* < 0.001		*p* < 0.001		*p* < 0.001		*p* < 0.001	
*CD68*	n	67	36	67	36	67	92	67	92
	Mean	3.31	9.46	2.32	7.31	1.85	10.58	1.37	4.72
	SD ±	3.04	3.98	2.60	3.72	1.87	9.08	1.69	4.42
	p-value	*p* < 0.001		*p* < 0.001		*p* < 0.001		*p* < 0.001	
*CD163*	n	73	47	73	47	68	95	68	96
	Mean	5.36	9.06	3.72	6.23	3.89	6.98	2.26	3.87
	SD ±	4.41	7.93	3.85	7.05	3.55	8.55	2.59	5.08
	p-value	*p* = 0.002		*p* = 0.022		*p* = 0.047		*p* = 0.096	
*CD11c*	n	58	44	58	44	75	95	75	96
	Mean	2.50	3.89	1.56	2.23	1.88	2.30	1.23	2.61
	SD ±	3.26	3.55	2.42	2.29	2.43	4.78	1.95	3.19
	p-value	*p* < 0.001		*p* = 0.002		*p* < 0.001		*p* < 0.001	

Values represent the number of cases (n), mean, standard deviation (SD), and p-value (Mann-Whitney U test) of marker expression in basal cell carcinoma (BCC) and cutaneous squamous cell carcinoma (cSCC). Significant (*p* ≤ 0.05) and highly significant values (*p* ≤ 0.001) are highlighted in bold lettering. The tumor core is comprised of mostly epithelial tissue with some stromal tissue, whereas the invasive front represents the transition zone from tumor to stroma tissue. Stroma labeling indices (sLI) contain cell counts of stromal cells within the invasive front or the tumor core. Total cell labeling indices (LI) contain cell counts of both tumor epithelial and stromal cells within the invasive front or the tumor core. Indices are expressed as a percentage. P-values were generated using the Mann-Whitney U test.

To summarize, all markers showed higher values in cSCC, as compared to BCC for both the invasive front and the tumor core, regardless of the index used for comparison.

#### Ratios of Foxp3/CD8, Foxp3/CD4, CD163/CD68, CD11c/CD68 and CD163/CD11c in BCC and cSCC by tumor core LI

**Fig. 4:**
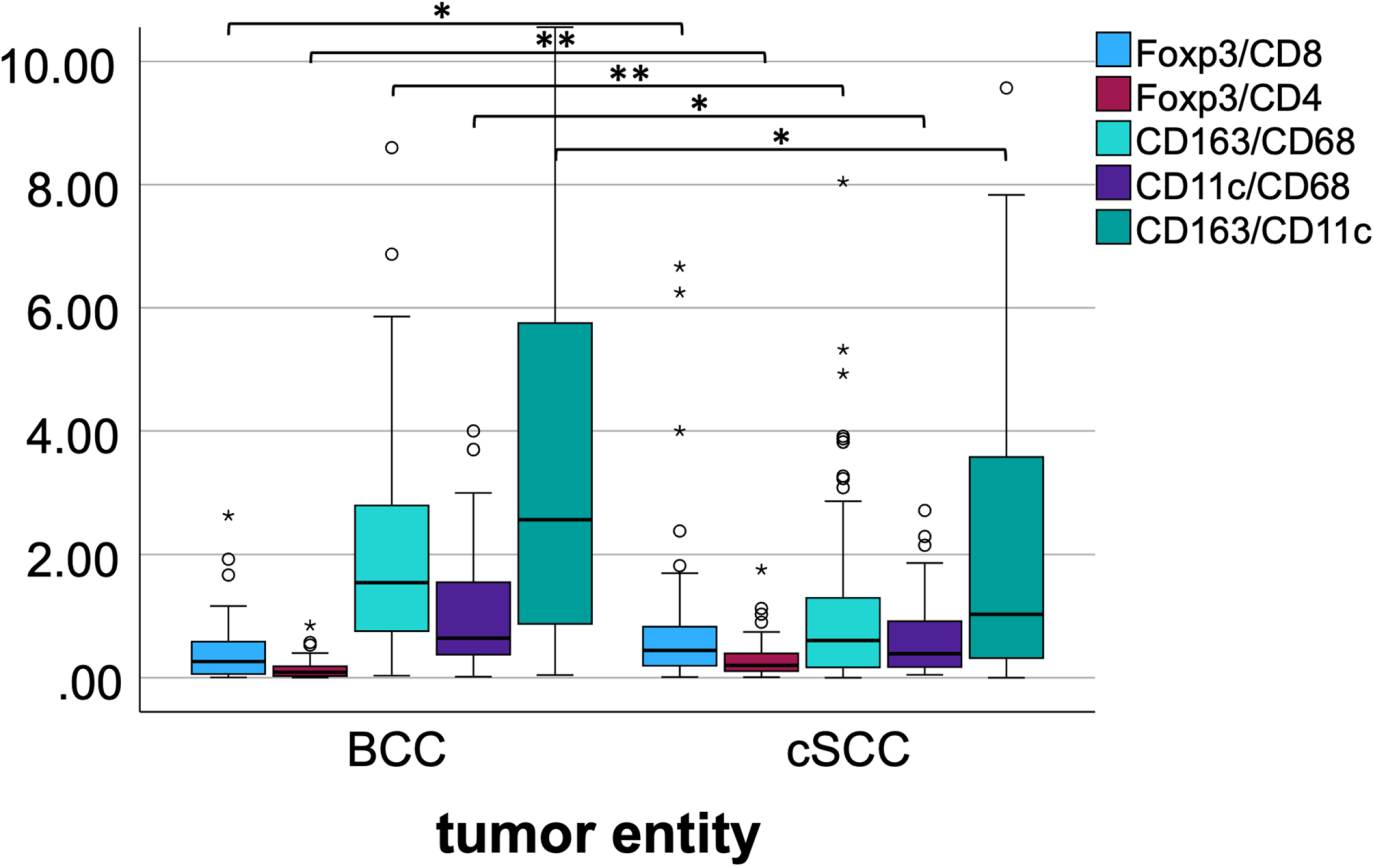
The box plots show ratios of Foxp3/CD8, Foxp3/CD4, CD163/CD68, CD11c/CD68 and CD163/CD11c in basal cell carcinoma (BCC) and cutaneous squamous cell carcinoma (cSCC) by labeling indices (LI) of the tumor core (TC). The tumor core is comprised of mostly epithelial tissue with some stromal tissue. LI contain cell counts of all detected cells within the TC. * = *p* < 0.050; ** = *p* ≤ 0.001. P-values were generated using the Mann-Whitney U test.

The ratio of Foxp3/CD8, and Foxp3/CD4 LI was significantly higher in the tumor core of cSCC as compared to BCC (p = 0.011, *p* < 0.001, respectively; Table 3; Fig. 4). Furthermore, significantly higher ratios were calculated for CD163/CD68 and CD11c/CD68 between cSCC and BCC (*p* < 0.001, *p* = 0.002, respectively; Table 3; Fig. 4) in favor of cSCC. Lastly, the CD163/CD11c ratio showed significant differences towards BCC, when compared to cSCC (*p* = 0.010; Table 3; Fig. 4) with means of 5.95 and 4.56 for BCC and cSCC, respectively (Table 3 4).

**Table 3:** Ratio comparison of Foxp3/CD8, Foxp3/CD4, CD163/CD68, CD11c/CD68, and CD163/CD11c mean, SD and p-values by total cell labeling indices.

Ratio		Tumor core total cell labeling index	
		BCC	cSCC
Foxp3 / CD8	n	56	77
	Mean	0.06	0.17
	SD	47.69	13.06
	p-value	*p* = 0.011	
Foxp3 / CD4	n	59	81
	Mean	0.03	0.10
	SD	70.7	18.95
	p-value	*p* < 0.001	
CD163 / CD68	n	62	91
	Mean	2.42	1.4
	SD	2.26	2.21
	p-value	*p* < 0.001	
CD11c / CD68	n	65	90
	Mean	1.39	0.98
	SD	1.39	1.83
	p-value	*p* = 0.002	
CD163 / CD11c	n	66	94
	Mean	5.95	4.56
	SD	18.07	11.69
	p-value	*p* = 0.010	

Values represent the number of cases (n), mean, standard deviation (SD), and p-value (Mann-Whitney U test) of marker ratios in basal cell carcinoma (BCC) and cutaneous squamous cell carcinoma (cSCC). Significant (*p* ≤ 0.05) and highly significant values (*p* ≤ 0.001) are highlighted in bold lettering. P-values were generated using the Mann-Whitney U test. The tumor core is comprised of mostly epithelial tissue with some stromal tissue. Total cell labeling indices (LI) of the tumor core contain cell counts of stromal and epithelial cells within the tumor core. These LI were divided for the individual marker pairings to calculate the ratios.

**Table 4:** Comparison of expression patterns between the invasive front and tumor core of CD4, CD8, Foxp3, CD68, CD163, and CD11c in BCC and cSCC by total cell labeling indices.

Marker		BCC		cSCC	
		Invasive front	Tumor core	Invasive front	Tumor core
*CD4*	n	79	80	51	87
	Median	8.86	5.45	13.09	8.17
	SD ±	7.57	7.47	7.14	9.47
	p-value	*p* = 0.008		*p* = 0.025	
*CD8*	n	70	73	49	83
	Median	4.12	1.80	8.41	4.86
	SD ±	8.22	3.94	6.72	7.11
	p-value	*p* < 0.001		*p* = 0.012	
*Foxp3*	n	68	60	48	91
	Median	0.72	0.34	2.06	1.69
	SD ±	0.78	0.82	1.69	2.11
	p-value	*p* = 0.012		*p* = 0.336	
*CD68*	n	67	67	36	92
	Median	1.58	0.78	5.87	3.67
	SD ±	2.60	1.69	3.72	4.32
	p-value	*p* = 0.002		*p* < 0.001	
*CD163*	n	73	68	47	96
	Median	2.67	1.64	4.37	2.02
	SD ±	3.85	2.59	7.05	5.08
	p-value	*p* = 0.004		*p* = 0.004	
*CD11c*	n	58	75	44	96
	Median	0.55	0.60	1.50	1.08
	SD ±	2.42	1.95	2.29	3.19
	p-value	*p* = 0.656		*p* = 0.316	

Comparison of marker expression in the invasive front versus the tumor core in basal cell carcinoma (BCC) and cutaneous squamous cell carcinoma (cSCC). Number of cases (n) and standard deviation (SD). The tumor core is comprised of mostly epithelial tissue with some stromal tissue, whereas the invasive front represents the transition zone from tumor to stroma tissue. Total cell labeling indices (LI) of the TMA core contain cell counts of stromal and epithelial cells within core. Indices are expressed as a percentage. P-values were generated using the Mann-Whitney U test.

In conclusion, Foxp3 positive cells relative to CD8 and CD4 demonstrate a higher abundance in cSCC compared to BCC, whereas both CD163 and CD68 exhibit a higher presence in BCC relative to CD68.

#### Comparison of expression patterns between the invasive front and tumor core of CD4, CD8, Foxp3, CD68, CD163, CD11c in BCC and cSCC

Comparative analysis was conducted on the differences in expression patterns by tumor compartment within BCC and cSCC, utilizing total cell LI. Significant differences were observed in the expression levels of CD4, CD8, CD68, and CD163 in both BCC and cSCC (*p* < 0.05; Table  4 ), with higher levels detected at the invasive front in comparison to the tumor core. For Foxp3, higher expression levels were observed in BCC and cSCC, with a greater abundance at the invasive front. However, a significant difference was only observed in BCC (*p* = 0.012; Table  4 ). No significant differences in expression levels were observed for CD11c (Table  4 ). For additional reference, Supplementary Fig. 1 accompanies the online article and visualizes data from Table  4. in additional boxplots comparing the expression patterns of the invasive front and the tumor core of BCC and cSCC, respectively.

#### Clinicopathological data in relation to CD4, CD8, Foxp3, CD68, CD163 and CD11c by sLI in cSCC

The clinicopathological data were subjected to a comparative analysis utilizing sLI, calculated from the marker’s expression in cSCC. A comparison of TNM-staged T1 to T2-T4 cases revealed that, only CD4 demonstrated a statistically significant difference, with a higher CD4 sLI observed in T1 cases compared to CD4 sLI in T2-T4 cases (*p* = 0.011; Table 5). When comparing TNM-classified cases with and without nodal infiltration (N+, N0, respectively) a significant result was obtained for CD11c with an elevated sLI in the N + group, compared to the N0 group (*p* = 0.041; Table 5). Furthermore, the cSCC cases were divided into two groups based on the depth of infiltration: those with infiltration depths greater than or equal to 6 mm and those with infiltration depths of less than 6 mm. In this comparison, CD68 sLI was higher in the group with an infiltration depth of ≥ 6 mm (*p* = 0.018; Table 5). Lastly, a comparison was conducted between cases where a previous malignancy was recorded and those where no previous malignancy was recorded. In this context, cases without previous tumors exhibited higher CD8 and CD11c sLI (*p* = 0.006, *p* = 0.029, respectively; Table 5). In conclusion, the extent of NMSC may influence both macrophage-like marker’s and T cell-marker’s sLI. Additionally, cSCC following previous tumor may alter marker expression profiles.

**Table 5:** Clinicopathological data comparison of CD4, CD8, Foxp3, CD68, CD163, and CD11c in cSCC by stroma labeling indices of the tumor core.

Tumor core sLI		pT	*n*	Median	SD	*p*-value	pN	*n*	Median	SD	*p*-value
cSCC	CD4	T1	41	18.10	± 10.5	**0.011**	N0	31	14.46	± 9.3	**0.626**
		T2-T4	35	12.33	± 9.4		N+	8	13.56	± 13.4	
		Total	76	14.72	± 10.4		Total	39	14.04	± 10.1	
	CD8	T1	38	8.74	± 11.2	**0.960**	N0	32	9.97	± 10.4	**0.685**
		T2-T4	34	10.79	± 7.3		N+	8	5.82	± 9.6	
		Total	72	9.36	± 9.6		Total	40	9.36	± 10.1	
	Foxp3	T1	39	3.93	± 4.7	**0.940**	N0	37	3.98	± 4.0	**0.656**
		T2-T4	39	4.70	± 3.8		N+	8	5.28	± 4.3	
		Total	78	4.55	± 4.3		Total	45	4.52	± 4.0	
	CD68	T1	41	8.86	± 7.6	**0.108**	N0	36	8.78	± 7.9	**0.233**
		T2-T4	38	13.14	± 10.5		N+	9	15.59	± 10.4	
		Total	79	10.13	± 9.3		Total	45	10.13	± 8.5	
	CD163	T1	41	3.21	± 9.2	**0.444**	N0	39	3.97	± 9.5	**0.166**
		T2-T4	40	4.95	± 5.6		N+	9	7.44	± 4.1	
		Total	81	4.42	± 7.6		Total	48	5.02	± 8.7	
	CD11c	T1	42	2.13	± 4.4	**0.282**	N0	37	2.89	± 3.9	**0.041**
		T2-T4	40	3.41	± 4.7		N+	8	8.58	± 6.9	
		Total	82	2.70	± 4.5		Total	45	3.29	± 4.9	
		Infiltration depth	n	Median	SD	p-value	Previous tumor	n	Median	SD	p-value
	CD4	< 6 mm	32	19.20	± 10.9	**0.056**	None	33	16.53	± 12.0	**0.274**
		≥ 6 mm	17	11.65	± 10.7		Previous	54	15.23	± 10.1	
		Total	49	15.64	± 11.0		Total	87	15.50	± 10.9	
	CD8	< 6 mm	30	10.68	± 11.6	**0.180**	None	29	14.66	± 8.2	**0.006**
		≥ 6 mm	18	8.27	± 6.8		Previous	53	7.62	± 9.4	
		Total	48	8.97	± 10.2		Total	82	9.61	± 9.2	
	Foxp3	< 6 mm	32	4.85	± 4.6	**0.888**	None	36	5.23	± 4.4	**0.319**
		≥ 6 mm	22	4.94	± 4.6		Previous	54	3.90	± 3.9	
		Total	54	4.85	± 4.5		Total	90	4.64	± 4.1	
	CD68	< 6 mm	31	7.13	± 5.6	**0.018**	None	36	8.78	± 9.2	**0.654**
		≥ 6 mm	23	13.45	± 11.2		Previous	56	9.19	± 9.1	
		Total	54	9.27	± 9.1		Total	92	8.78	± 9.1	
	CD163	< 6 mm	33	2.84	± 8.9	**0.283**	None	38	4.90	± 4.8	**0.951**
		≥ 6 mm	23	5.13	± 8.3		Previous	56	3.91	± 10.4	
		Total	56	3.59	± 8.6		Total	94	4.36	± 8.6	
	CD11c	< 6 mm	34	2.34	± 4.2	**0.537**	None	36	4.22	± 5.1	**0.029**
		≥ 6 mm	23	3.37	± 5.1		Previous	58	1.90	± 4.4	
		Total	57	2.53	± 4.5		Total	94	2.13	± 4.8	

Clinicopathological data comparison of CD4, CD8, Foxp3, CD68, CD163, and CD11c by stroma labeling indices in cutaneous squamous cell carcinoma (cSCC). Stroma labeling indices (sLI) include only cell counts from stromal cells within the tumor core. N represents the total number of cases. The tumor core is comprised of mostly epithelial tissue with some stromal tissue. P-values were generated using the Mann-Whitney U test.

Further visualization of the results, can be found as supplementary material accompanying the online article. The supplementary material section contains Spearman correlation coefficient values and two-sided adjusted p-values in the Supplementary Tables 1 and 2, as well as additional boxplots comparing the expression patterns of the invasive front and the tumor core of BCC and cSCC, respectively, in the Supplementary Fig. 1 based on the results shown in Table 3.

## Discussion

This study built on our group’s prior research on immune cell infiltration in BCC and cSCC^11,12^ and employed a semiquantitative approach to investigate CD4, CD8, Foxp3, CD68, CD163, and CD11c markers associated with different T cells and macrophage polarization in BCC and cSCC. Despite the relatively low overall mortality, the high number of reported incident cases resulted in NMSC-related deaths totaling approximately 63,731 in 2020, which was comparable to the number of cancer-related deaths of melanoma of the skin (57,043) or cancer located at the gallbladder (84,695)^28^. Consequently, there is a clear need to enhance our understanding of the TME in both entities, with a particular focus on lymphocytes and monocyte-derived cells, to facilitate the development of novel therapeutic approaches for the management of these malignancies.

The TME exhibits considerable heterogeneity, resulting in significant variations in tumor characteristics among individuals^29^, even though by TNM-classification the tumor may appear similar. In addition, the immune contexture, influenced by factors such as density, composition, functional state, and organization of the leukocyte infiltrate within the tumor, can provide prognostic information and predict treatment response^30^. Our results highlight a relevant infiltration of all investigated immune cell markers at the invasive margin, as well as the tumor core. This indicates potential tumorbiological relevance of both T cell and macrophage populations in BCC and cSCC within their respective TMEs. In addition, previous findings from our group also indicated a relevant presence of activating and inhibitory checkpoint markers, PD-1/PD-L1 and CD28/CD86, within these NMSC TMEs^11^. Furthermore, when significant differences were observed, the invasive front was the predominant site of immune cell localization within the tumor entities. This expression profile also corresponds with the results of our workgroup’s investigation of immune-checkpoint expression in both BCC and cSCC, where the invasive front also emerged as the predominant site of detected signal. Moreover, higher infiltrate values were predominantly observed in cSCC compared to BCC^11,12^.

Relevant immune cell presence has been described to influence the response to immunotherapy in the treatment of solid malignancies, among others^31^. For many malignancies, challenges remain regarding the actual response rates, as only a minority of patients respond well to immunotherapy^32^. For the majority of solid tumors, response rates range from 15 to 30%^3,33^. A comparison of these response rates with those observed in BCC and cSCC reveals that, in general, NMSC seems to exhibit a superior response rate. However, even response rates of 51% in metastatic or locally advanced cSCC are unsatisfactory, as 49% of patients still remain unresponsive^34^. In addition, immune checkpoint inhibitors appear to be more effective in tumors with high inflammatory signatures^29^. Meanwhile, there seems to be negligible benefit from this treatment modality in tumors exhibiting low inflammatory signatures^29^. This seems interesting, as our results show a lower lymphocyte expression for the investigated markers in BCC, when compared to cSCC. However, in cases of locally advanced or metastatic BCC, response rates of 6–31% have been reported after anti-PD1 therapy^35^.

In a systematic review, Presti et al. supported the hypothesis, that tumor infiltrating lymphocytes (TILs) should play a role in predicting the response of solid malignancies, including cSCC, to ICI-IT, since they are the main effectors of antitumoral activity^36,37^. In the context of head and neck squamous cell carcinoma, higher levels of CD3+, CD8+, and Foxp3 + TILs have been associated with a more favorable prognosis^36,38^. Furthermore, in a study by Schulze et al., results indicate that in non-small cell lung cancer, strong CD4 infiltration is associated with higher tumor stages^39^. Conversely, our findings indicate, that higher CD4 expression may be linked to T1 cSCC rather than T2-4 carcinomas. However, more evidence linking CD4 expression to T-staging in NMSC and cSCC is limited.

In oral squamous cell carcinoma, Santos Pereira et al. reported of a significant association between higher levels of CD8 + T cells and the absence of metastasis^40^. Our data also show higher, although insignificant, CD8 values for N0 cases, when compared to N + cases. However, due to the small number of N + cases in our patient cohort, limited conclusions can be drawn from these results and further research is needed.

Cunha et al. postulated, that similarities in the T cell fingerprint within the TME of BCC and cSCC indicate a similar host response^41^. In our analysis, the percentages of CD8, CD4 and Foxp3 expression varied between BCC and cSCC, but their trends remained similar. Furthermore, our results show an increased expression of CD4 over CD8 in BCC and cSCC, as well as an overall increased expression in cSCC compared to BCC. The increased frequencies of CD4 over CD8 are also in agreement with previous results of Cunha et al.^41^. In both colorectal and ovarian cancer, increased numbers of CD8 TILs are correlated with improved survival^42^, highlighting their potential anti-tumor effects.

The interpretation of CD4 expression profiles develops to be increasingly complex because of the variety of biological features within the CD4 + T cell lineage^43^. This plasticity of CD4 + T cells leads to the possibility of facilitating or hindering an effective anti-tumor response^38^. On the other hand, T cells expressing the surface protein CD8, are classified as effector T cells or cytotoxic T cells, which have the ability to induce apoptosis in tumor cells^42^. However, there are also CD8 + Tregs that appear to have enhanced regulatory properties compared to CD4 + Tregs in cSCC^8^.

Tregs are more abundant in BCC and cSCC, as compared to healthy skin^8^. Since they are not known to proliferate within the tumor, their recruitment from the bloodstream seems likely^8^. In squamous cell carcinoma (SCC), Clark et al. hypothesized, that immune evasion is achieved in part due to Treg recruitment^44^. Furthermore, treatment of SCC with Imiquimod resulted in a reduction of Treg presence to a level seen in healthy skin, thus potentially reducing their pro-tumoral effect^44^. However, implications of imiquimod’s effect on the Foxp3 landscape within the BCC TME, to our knowledge, have not been made. In our study, Tregs were present in both our BCC and cSCC samples, with higher numbers in cSCC compared to BCC. Notably, our findings did not suggest a high Foxp3 to CD4 ratio, as previously described by Omland et al., where approximately 45% of CD4 + T cells in BCC were Tregs^45^. A comparison between the results seems difficult as only a small number of patient samples were analyzed by Omland et al. (*n* = 19) and no further characteristics of the patient population were given^45^. Further, there is also evidence from RNA sequencing data, which did not observe increased Foxp3 levels within the BCC TME^46^.

In both BCC and cSCC we observed higher levels of CD163 expression, when compared to CD68 and CD11c. This indicates that an M2-like macrophage polarization is more prominent in both microenvironments. This finding is consistent with previously described findings of a predominance of M2-like macrophages over other macrophages in both BCC and cSCC^47,48^. Furthermore, when comparing the ratio of CD163 to CD68, and CD163 to CD11c between BCC and cSCC, BCC showed significantly more CD163 expression compared to both CD68 and CD11c when contrasted with cSCC. However, when comparing the labeling indices of CD163 between BCC and cSCC, higher values were observed for cSCC compared to BCC. In general, a TME with a dominance of M2-like macrophages over other macrophage types is considered to be a pro-tumor TME that facilitates tumor progression and inhibits a strong T cell mediated response^49^. In addition, M2-like macrophages have a poor antigen presenting capacity, which further exacerbates their negative impact on an anti-tumor response^50^. Finally, conflicting results have been reported regarding M2-like macrophage levels and their link to BCC recurrence^51^. In the study conducted by Beksaç et al., no correlation was identified between the levels of M2-like macrophages in patients with BCC who experienced recurrence^51^. Conversely, the findings reported by Tjiu et al. indicated that elevated levels of M2-like macrophages were associated with more aggressive behavior in BCC^51^. Consequently, further research is necessary to ascertain the role of the predominant M2-like macrophage environment in the recurrence of BCC.

Finally, most patients are concerned about secondary tumors or recurrence after their initial battle with cancer. Secondary tumors, or secondary primary malignancies (SPM), are defined as tumors that develop after a primary cancer and are unrelated to the primarius. A statistical analysis of cancer survivors in the United States between 1992 and 2008 found that nearly 1 in 12 patients diagnosed with a common cancer developed a SPM^52^. In addition, more than half of these patients died from their SPM^52^. However, to our knowledge, there are no data comparing the expression of T cell- and macrophage-associated markers in SPM cSCC with the occurrence of previous primary malignancies. Here, our data show a significant increase in sLI of the tumor core for CD8 and CD11c in cases without prior primary malignancies compared to those who suffered from a primary tumor. This may suggest that either the long-term effects of the primary cancer or its treatment may have an effect on SPM-TME composition. An alternative hypothesis to be considered is that these patients exhibit a diminished CD8 response in general. However, further research is needed to validate and explore possible mechanisms behind these findings.

### Limitations of the study

Limitations of this study were similar to those previously described^11^. The combination of both TMA and robust singleplex IHC staining resulted in reliable results and the ability to investigate a larger patient cohort. However, certain specimen features might have been lost due to the nature of the TMA core sampling from paraffin-embedded resection tissue. Further, due to the use of one TMA slide per marker, the lack of tissue abundance limited the analysis of the full study cohort in some instances. Additionally, the utilization of the well-established singleplex IHC protocol lacks the ability to colocalize markers and/or their spatial analysis, when compared to a multiplex approach. Nonetheless, it is this author’s opinion that the methodical approach of employing singleplex IHC of TMA was sufficient to provide preliminary insight into the expression patterns of T cell- and macrophage-associated markers within BCC and cSCC.

## Conclusion

This study offers a comprehensive analysis of the TME of both BCC and cSCC, emphasizing the distinct expression patterns of their respective TME with regard to T cell- and macrophage-associated markers. The analysis revealed comparable trends in the T cell fingerprint among the two types of cancer. However, the cSCC TME exhibited higher expression levels of all investigated markers when compared to BCC. Furthermore, the predominant site of marker expression was observed at the invasive front, contrasting with the tumor core. A comprehensive analysis of these TMEs, probing their pro- and anti-inflammatory characteristics, is imperative to draw definitive conclusions regarding their impact on clinicopathological manifestations, response to IT, and predictive capabilities.

## Supplementary Information

Below is the link to the electronic supplementary material.

Supplementary Material 1

## Data Availability

All data generated or analyzed during this study may be provided by the authors. Further inquiries can be directed to the corresponding author.

## Code availability

The custom Python script, which was written to accurately match each Qupath result with the corresponding dataset in SPSS through .csv files, may be obtained upon request from the corresponding author.
