# Supplementary material for: Comparative profiling of T cell and macrophage subsets in cutaneous squamous cell carcinoma and basal cell carcinoma
Source: Sci Rep. 2025 Oct 9;15:35240. doi: 10.1038/s41598-025-22486-1 (PMC12511370; doi:10.1038/s41598-025-22486-1)
Supplement: Supplementary file 1 — Supplementary Material 1 [file 41598_2025_22486_MOESM1_ESM.docx]

**Supplementary Material:**

**Tables:**

| **BCC tumor core sLI** | | CD4 | CD8 | Foxp3 | CD68 | CD163 | CD11c |  |
| --- | --- | --- | --- | --- | --- | --- | --- | --- |
|  |  |  |  |  |  |  |  |  |
| CD4 | rho (ρ) | 1000 | .649^**^ | .362^**^ | .449^**^ | .536^**^ | .423^**^ |  |
|  | p-value | . | <.001 | 0.004 | <.001 | <.001 | <.001 |  |
|  | n | 80 | 73 | 60 | 67 | 68 | 75 |  |
| CD8 | rho (ρ) | .649^**^ | 1000 | 0 | .271^*^ | .389^**^ | .315^**^ |  |
|  | p-value | <.001 | . | 0.086 | 0.03 | 0.001 | 0.007 |  |
|  | n | 73 | 74 | 58 | 64 | 65 | 71 |  |
| Foxp3 | rho (ρ) | .362^**^ | 0.227 | 1000 | .459^**^ | .427^**^ | .375^**^ |  |
|  | p-value | 0.004 | 0.086 | . | <.001 | 0.001 | 0.003 |  |
|  | n | 60 | 58 | 61 | 52 | 54 | 59 |  |
| CD68 | rho (ρ) | .449^**^ | .271^*^ | .459^**^ | 1000 | .571^**^ | .523^**^ |  |
|  | p-value | <.001 | 0.03 | <.001 | . | <.001 | <.001 |  |
|  | n | 67 | 64 | 52 | 67 | 62 | 65 |  |
| CD163 | rho (ρ) | .536^**^ | .389^**^ | .427^**^ | .571^**^ | 1000 | .337^**^ |  |
|  | p-value | <.001 | 0.001 | 0.001 | <.001 | . | 0.005 |  |
|  | n | 68 | 65 | 54 | 62 | 69 | 68 |  |
| CD11c | rho (ρ) | .423^**^ | .315^**^ | .375^**^ | .523^**^ | .337^**^ | 1000 |  |
|  | p-value | <.001 | 0.007 | 0.003 | <.001 | 0.005 | . |  |
|  | n | 75 | 71 | 59 | 65 | 68 | 76 |  |

Supplementary table 1. Spearman correlation of CD4, CD8, Foxp3, CD68, CD163 and CD11c in basal cell carcinoma (BCC). Stroma cell labeling indices (sLI) contain cell counts of both tumor epithelial and stromal cells within the tumor core. Rho represents the correlation coefficient and p-values represent significance levels of the two-tailed spearman correlation. N represents the total number of cases.

| **cSCC tumor core sLI** | | CD4 | CD8 | Foxp3 | CD68 | CD163 | CD11c |  |
| --- | --- | --- | --- | --- | --- | --- | --- | --- |
|  |  |  |  |  |  |  |  |  |
| CD4 | rho (ρ) | 1000 | .340^**^ | .374^**^ | -.232^*^ | 0.146 | 0.115 |  |
|  | p-value | . | 0.002 | <.001 | 0.039 | 0.197 | 0.308 |  |
|  | n | 87 | 77 | 80 | 79 | 80 | 81 |  |
| CD8 | rho (ρ) | .340^**^ | 1000 | .323^**^ | 0.009 | -0.12 | .286^*^ |  |
|  | p-value | 0.002 | . | 0.005 | 0.937 | 0.3 | 0.011 |  |
|  | n | 77 | 82 | 75 | 76 | 77 | 79 |  |
| Foxp3 | rho (ρ) | .374^**^ | .323^**^ | 1000 | 0 | -0.033 | 0.088 |  |
|  | p-value | <.001 | 0.005 | . | 0.994 | 0.766 | 0.426 |  |
|  | n | 80 | 75 | 90 | 83 | 86 | 85 |  |
| CD68 | rho (ρ) | -.232^*^ | 0.009 | 0.001 | 1000 | 0 | .411^**^ |  |
|  | p-value | 0.039 | 0.937 | 0.994 | . | 0.085 | <.001 |  |
|  | n | 79 | 76 | 83 | 92 | 89 | 89 |  |
| CD163 | rho (ρ) | 0.146 | -0.12 | -0.033 | 0.184 | 1000 | 0 |  |
|  | p-value | 0.197 | 0.3 | 0.766 | 0.085 | . | 0.096 |  |
|  | n | 80 | 77 | 86 | 89 | 94 | 90 |  |
| CD11c | rho (ρ) | 0.115 | .286^*^ | 0.088 | .411^**^ | 0.177 | 1000 |  |
|  | p-value | 0.308 | 0.011 | 0.426 | <.001 | 0.096 | . |  |
|  | n | 81 | 79 | 85 | 89 | 90 | 94 |  |

Supplementary table 2. Spearman correlation of CD4, CD8, Foxp3, CD68, CD163 and CD11c in cutaneous squamous cell carcinoma (cSCC). Stroma cell labeling indices (sLI) contain cell counts of both tumor epithelial and stromal cells within the tumor core. Rho represents the correlation coefficient and p-values represent significance levels of the two-tailed spearman correlation. N represents the total number of cases.


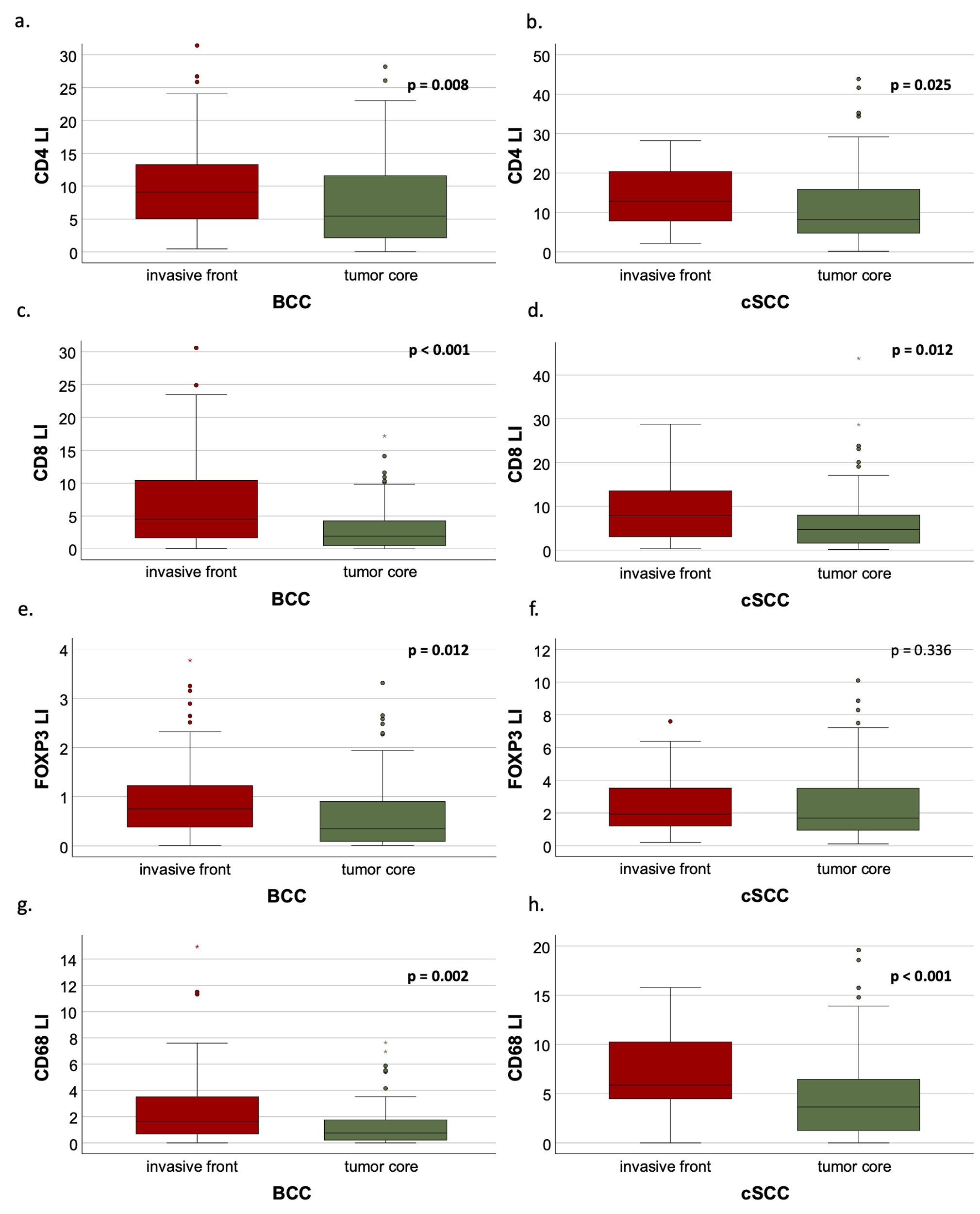


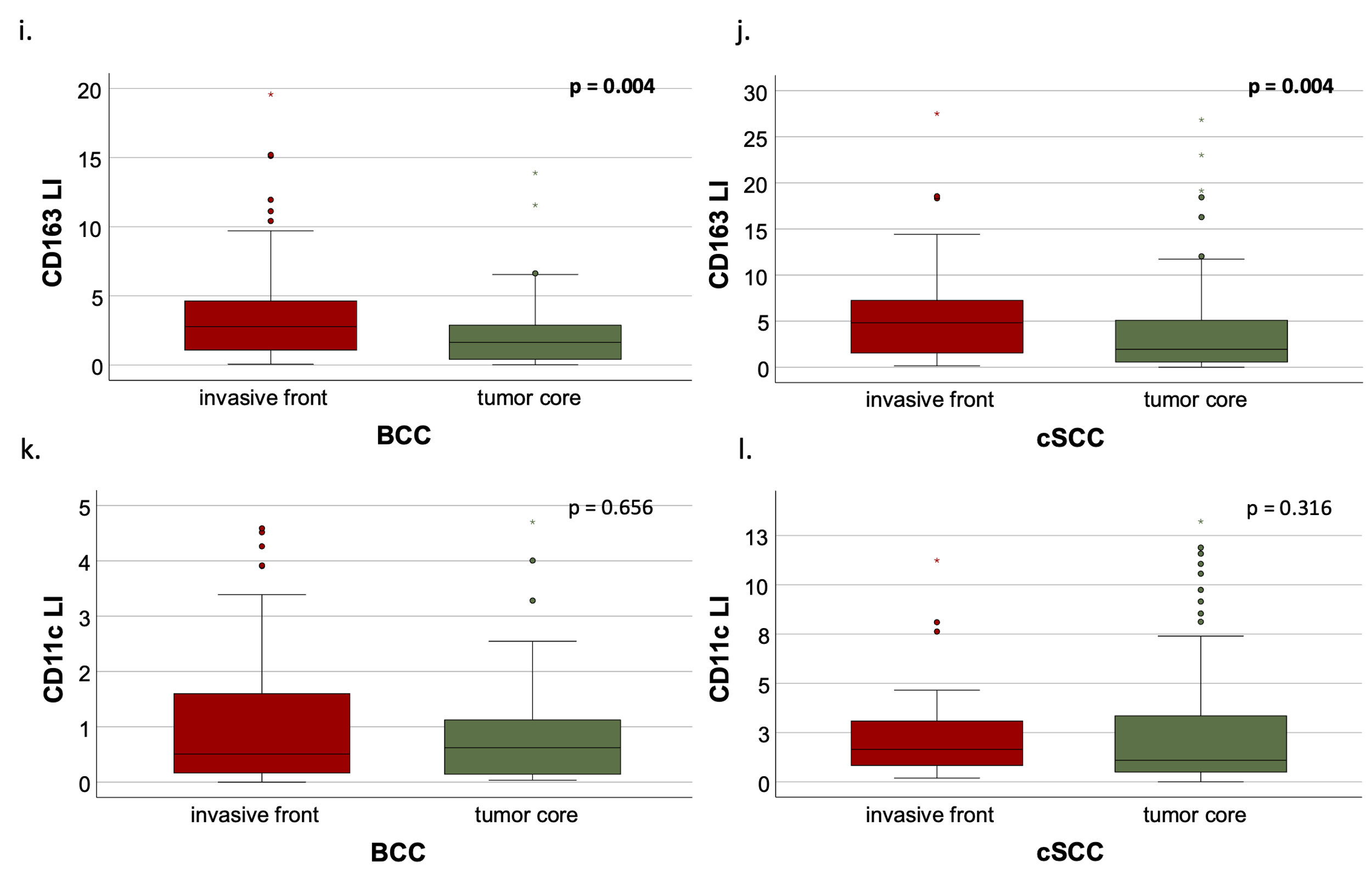


Supplementary Figure 1. Comparison of expression patterns in the invasive front and tumor core of CD4, CD8, Foxp3, CD68, CD163, and CD11c in BCC and cSCC by stroma labeling indices. **(a-l)**: The boxplots show a comparison of marker expression in the invasive front versus the tumor core of CD4, CD8, Foxp3, CD68, CD163, and CD11c in basal cell carcinoma (BCC) and cutaneous squamous cell carcinoma (cSCC). The tumor core is comprised of mostly epithelial tissue with some stromal tissue, whereas the invasive front represents the transition zone from tumor to stroma tissue. Stroma labeling indices (sLI) contain cell counts of stromal cells within the invasive front or the tumor core. Indices are expressed as a percentage. P-values were generated using the Mann-Whitney U test.
